# Enhanced antibacterial metabolite production through the application of statistical methodologies by a *Streptomyces nogalater* NIIST A30 isolated from Western Ghats forest soil

**DOI:** 10.1371/journal.pone.0175919

**Published:** 2017-04-24

**Authors:** Jubi Jacob, Reshma Uma Rajendran, Syama Hari Priya, Jayamurthy Purushothaman, Dileep Kumar Bhaskaran Nair Saraswathy Amma

**Affiliations:** 1 Agro- Processing and Technology Division, CSIR-National Institute for Interdisciplinary Science and Technology (NIIST), Thiruvananthapuram, Kerala, India; 2 Academy of Scientific and Innovative Research (AcSIR),CSIR-National Institute for Interdisciplinary Science and Technology (NIIST), Thiruvananthapuram, Kerala, India; Universite Paris-Sud, FRANCE

## Abstract

*Streptomyces* strains isolated from Nelliyampathy forest soil of Western Ghats, Kerala, India were evaluated for their antibacterial efficacy against two indicator pathogenic bacteria (*Escherichia coli* and *Staphylococcus aureus*). Among 140 strains tested, sixteen recorded potent antibacterial properties and were further screened against eleven bacterial pathogens. A strain identified as *Streptomyces nogalater* and designated as NIIST A30 exhibited maximum inhibition against all the test pathogens. Among the eight fermentation media tested, inorganic salts starch broth recorded the best for antibacterial production. The ethyl acetate crude extract exhibited antioxidant properties with IC_50_ value of 30 μg/mL and had no cytotoxicity towards L6, H9c2 and RAW 264.7 cell lines up to a concentration of 50 μg/mL. Maximum metabolite production was achieved in pH 7.0 at 35°C after 7 days incubation. The significant media components for maximum metabolite production were optimized through response surface methodology employing Plackett-Burman and Box-Behnken designs. The composition of the final optimized medium was soluble starch, 14.97g; (NH_4_)_2_SO_4_, 2.89g; K_2_HPO_4_, 2.07g; MgSO_4_.7H_2_O, 1g; NaCl, 1g, CaCO_3_, 2g; FeSO_4_.7H_2_O, 1mg; MnCl_2_.7H_2_O, 1mg; and ZnSO_4_.7H_2_O, 1mg per litre of distilled water. The optimization resulted an antibacterial activity of 28±1.5mm against *S*. *epidermidis* which was in close accordance with the predicted value of 30 mm. It is also evident from the result that an increase of 86.66% antibacterial production was recorded in optimized media. The chosen method was economical, efficient and useful for future antibacterial drug discovery from a broad spectrum metabolite producer like *Streptomyces nogalater* NIIST A30.

## Introduction

The demand and search for novel antibiotics is increasing due to the emergence of multiple drug resistance in pathogens around the globe associate with the discovery of very less number of new potential molecules. For this, *Streptomyces* species are one of the major targets for mining new antibiotics. The history of antibiotics from these filamentous, Gram-positive bacteria got attention among the researchers with the discovery of streptothricin in 1942 followed by streptomycin after two years [1, 2]. The genus *Streptomyces* belonging to the group actinobacteria whose species are widely distributed in terrestrial and marine habitats, have been regarded as a bowl of natural products with various interesting biological activities including antibacterial properties. The metabolites include antibiotics, antitumor agents, immunosuppressants, antihelminthics and plant growth hormones [3].By employing many improved techniques under various screening strategies, the rate of discovery of natural products exceeded to a million so far, out of which 22,250 bioactive compounds are from microorganisms in which 45% are produced by actinobacteria, 38% by fungi and 17% from other bacteria. It was also recorded that among the actinobacteria, *Streptomyces* species produces nearly 80% of all the reported metabolites [4].

A major chunk of natural products from bacterial origin have been identified from organisms that inhabit the soil. Since soil itself is a highly porous mixture of minerals and organic matter, it has been shown that filamentous bacteria might be able to bridge air- filled gaps between soil particles better than their unicellular counterparts. The genus actinomycetes possess an outstanding position as targets in various screening programs for their proven ability to produce novel metabolites viz. antibiotics and other lead molecules of pharmaceutical interest. Currently, various scientific studies have proven their biological properties such as antimicrobial, antioxidant, anti-inflammatory and anti-cancer properties [5].

The composition of a fermentation medium is a significant factor that influences the level of antimicrobial production by a microorganism [6].The source and concentration of nutrients along with the cultural conditions are known to have mysterious effects on bioactive metabolite production in *Streptomyces* too, since the antibiotic production is not a static property [7]. Therefore, designing an appropriate medium and conditions for cultivation has prime importance in improving the antibiotic yield [8].Single parameter per trial used in conventional media optimization technique is a laborious and time consuming process involving a large number of experimental trials to determine the optimum levels of all variables. In addition, it may not provide the correlation between different parameters and often fails to identify the variables that give an optimum response. A viable alternative for this is to develop a strategy which involves statistical optimization that can resolve those issues related to conventional optimization [9].

Response surface methodology (RSM), a collection of mathematical and statistical techniques for building empirical models, has been recognized as a fascinating approach for enhanced production of commercially important bioactive metabolites and enzymes. It has been successfully applied in various facets viz. improvement of biofuel and biomass production [10], and identifying the factors enhancing enzyme production as well as assessment of carbon mineralization from sewage sludges [11].Hence in the present study, RSM was preferred to optimize the media components in culture medium for maximum antibacterial metabolite production.

In the present study, a strain designated as *Streptomyces nogalater* NIIST A30, was investigated for enhanced antibacterial metabolite production through RSM methodology with the application of Plackett-Burman design (PBD) and Box-Behnken design (BBD).

## Materials and methods

### Ethics statement

This is to confirm that the samples were collected from Nelliyampathy forest hills, a part of Western Ghats area where no specific permission was required for the collection. We also confirm that the present study did not involve any endangered or protected species.

### Chemicals and reagents

The chemicals and reagents used for microbiological work were procured from Hi-Media, Mumbai, India where as the solvents used for extraction and thin layer chromatography (TLC) were purchased from Merck, Mumbai, India. Normal cell lines such as L6 rat myoblast, H9c2 rat cardiac myoblast and RAW 264.7 murine macrophage were obtained from National Centre for Cell Sciences (NCCS), Pune, India.

### Site, sample collection and isolation of actinomycete strains

Soil samples were collected from Nelliyampathy forest hills (10°32’20.2ʺN 76°41’37.1ʺE) of the Western Ghats region in Kerala, India and brought to the laboratory under sterile conditions. The samples were then dried in an oven (Equitron, Mumbai, India) at 45°C and processed within 36 h for the isolation of actinomycete strains. For this, 10 g of soil samples were suspended in 90 mL sterile distilled water and shaken vigorously for 1 h in a shaking incubator (Lab Companion, Korea) at 160 rpm at 28±2°C. Samples were then allowed to settle and serial dilutions up to 10^−3^ were prepared and aliquot of 100 μl from each dilution was spread evenly on actinomycete isolation agar (AIA) and Potato dextrose Agar (PDA) plates in triplicates and incubated at 28±2°C for 14 to 28 days. The emerging colonies with different morphological characters were selected and the purified strains were maintained on AIA. The viability of the strains were checked in AIA, PDA and ISP4, where PDA was the best, hence further storage and maintenance of the culture was done in PDA.

### Test pathogens

The bacterial pathogens used for antibacterial studies were *Bacillus cereus* MTCC 1305, *B*. *subtilis* MTCC 2756, *Mycobacterium smegmatis* MTCC 993, *Staphylococcus aureus* MTCC 902, *S*. *epidermidis* MTCC *435*, *S*. *simulans* MTCC 3610, (all Gram positive) and *Escherichia coli* MTCC 2622, *Klebsiella pneumoniae* MTCC 109, *Proteus mirabilis* MTCC 425, *Pseudomonas aeruginosa* MTCC 2642, *Salmonella typhi* MTCC 3216, (all Gram negative). All the bacterial strains were procured from Microbial Type Culture Collection and Gene Bank (MTCC), CSIR-Institute of Microbial Technology (IMTECH), Chandigarh, India.

### The preliminary antibacterial studies using live *Streptomyces* species

The primary screening of 140 *Streptomyces* isolates was done by agar overlay technique. For this, *Streptomyces* isolates were spot inoculated on PDA plates and grown at 28±2°C for 7 days. Then the plates were covered with 0.6% of nutrient agar medium previously seeded individually with two test indicator bacterial pathogens (*Escherichia coli* and *Staphylococcus aureus*) to evaluate their antibacterial activity. The activity was recorded after 24 h of growth at 37°C and expressed as zone of inhibition (in mm).

In the secondary screening, 16 *Streptomyces* strains which exhibited significant antibacterial activity against the two indicator organisms were further tested against eleven bacterial pathogens by agar overlay method as mentioned above, to check the broad spectrum antibacterial properties. The strain which recorded best zone of inhibition was selected for further detailed studies.

### Morphological, cultural and physiological characterization

The strain NIIST A30,which recorded the best antibacterial activity in the screening as mentioned above was characterized on the basis of morphological features, including colony characteristics and pigment production on various media *viz*. Nutrient agar (NA); Bennet’s agar (BA); Czapek dextrose agar (CDA); Kustner’s agar (KA); *International Streptomyces Project* (ISP) agar such as ISP1 (Tryptone yeast extract agar), ISP 2 (Malt extract agar), ISP 3 (Oat meal agar), ISP4 (Inorganic salt starch agar), ISP 5 (Glycerol asparagine agar), ISP 6 (Peptone yeast extract agar) and ISP 7 (Tyrosine agar);Potato dextrose agar (PDA) and Starch casein agar (SCA). Colour formation was determined according to NBS/IBCC Colour System. (http://www.dodomagnifico.com/Colors/Cent.html).

Morphological characteristics of isolate NIIST A30 were assessed by light microscopy and scanning electron microscopy (SEM). For SEM analysis, sterilized circular aluminium stubs were inserted into the PDA plate at an angle of about 45°C and sterile coverslips were inserted at the same angle on the sample plate for viewing grown culture. The plates with stubs and coverslip were incubated at 37°C for 24 h to check any contamination during the handling procedure. After 24 h, isolate NIIST A30 was introduced along the line where the surface of the stub met the agar medium and incubated at 28±2°C for 7 days. The stubs were then carefully removed and coated under vacuum, with a film of gold for 15–20 minutes and viewed on the scanning electron microscope (Zeiss Evo 40 EP, Germany). Similarly, the cover slip was also mounted on the glass slide having one drop of methylene blue (0.3 g in 10 mL distilled water) and fixed slides were observed under light microscope (Olympus CX41, Japan).

Biochemical tests *viz*. Gram staining, catalase, oxidase, hydrolysis of cellulose, gelatin, lipids, pectin, protein, starch and urea were done using the standard procedures [12]. Antibiotic susceptibility of the strain was done using various antibiotic discs (Hi-Media, India). Cultural characteristics such as pH range (0.5 to 12) and temperature range (25 to 45°C)were done by inoculating the strain in inorganic salt starch broth. The utilization of carbon sources was determined by growing the isolate in Basal liquid medium (BLM) supplemented with 1% of various carbon sources (fructose, galactose, glycerol, lactose, maltose, mannitol, starch, sucrose, and xylose). BLM alone inoculated with the test strain served as a control. Nitrogen utilization of the strain was tested in BLM with 1% of various nitrogen sources *viz*. ammonium chloride, ammonium sulphate, beef extract, biopeptone, casein, malt extract, meat extract, meat infusion powder, meat peptone, peptone, potassium nitrate, soybean meal, urea and yeast extract. BLM supplemented with glucose served as a control [13]. All the results were noted after 7 days of incubation.

### Molecular identification of NIIST A30 using 16SrRNA sequencing and phylogenetic analysis

The total genomic DNA was isolated from NIIST A30 using NucleoSpin^®^ Tissue Kit (Macherey-Nagel). PCR amplification were carried out using 16S rRNA primers (forward primer, 16S-RS-F-5’CAGGCCTAACACATGCAAGTC3’ and reverse primer, 16S-RS-R- 5’GGGCGGWGTGTACAAGGC3’) and amplification profile consists of an initial denaturation at 95°C for 5 minutes followed by 35 amplification cycles of 95°C for 30 sec, 60°C for 40 sec and 72°C for 60 sec.

The PCR product was checked in 1.2% agarose gels and visualized in a UV transilluminator and the gel imaging was done using Gel documentation system. The amplified product was purified by ExoSAP-IT Treatment and sequenced in ABI 3730 DNA Analyzer (Applied Biosystems, USA).

### Sequence analysis and phylogenetic tree construction

The sequence quality was tested using Sequence Scanner Software v1 (Applied Biosystems, USA). Sequence alignment and required editing of the obtained sequences were carried out using Geneious Pro v5.6 [14].The high-quality 16S rRNA partial sequence was deposited in GenBank data library (Accession No. KX190774.1).The sequence was compared with other related species downloaded from the public database using EzTaxon tool [15] and a phylogenetic tree was constructed with MEGA version 6. Sequences were aligned using the computer package ClustalW and were analyzed to determine the relationships between isolates by the neighbor-joining method. Bootstrap values were generated using 1000 replicates.

### Standardization of fermentation medium for maximum antibacterial metabolite production

The strain was cultivated in various media such as Glucose soybean meal broth, Tryptone yeast extract broth, Yeast malt broth, Inorganic salt starch broth, Kuster’s broth, Nutrient broth, Sabouraud dextrose broth and Starch casein broth (see Table E in S1 File). All the media were inoculated with the strain NIIST A30 and incubated for 7 days at 28±2°C. After fermentation, the culture broth was centrifuged (Remi cooling centrifuge, Mumbai, India) at 10,000 rpm for 15 min to obtain cell free culture filtrate. The culture filtrate was then extracted with an equal amount of ethyl acetate and agitated for 45 min. The solvent layer was concentrated using a rotary evaporator (Buchi, Switzerland) at 40°C and the crude extract was collected by rinsing with 5 ml of methanol, dried, and were kept at 4°C for further studies.

### Antibacterial study of the crude extracts from different fermentation media

The antibacterial activity of the crude extract from different fermentation media was examined using agar disc diffusion method [16].The test bacteria was first inoculated into nutrient broth tubes and incubated at 37°C for 24 h. Each of the inoculums were then adjusted to 0.5 McFarland turbidity standards and swabbed into Mueller—Hinton agar (MHA) plates. Six mm sterile filter paper discs (Whatman No.3, Hi-Media, India) impregnated with ethyl acetate crude extract (25 μl) was placed on the swabbed MHA. A sterile disc with methanol served as the control. Plates were then incubated at 37°C and the zone of inhibition was measured (in mm) after 24 h incubation.

### Antioxidant activities of crude ethyl acetate extract (scavenging of free radicals)

#### DPPH free radical scavenging assay

The antioxidant potential of crude ethyl acetate extract was determined by free radical DPPH (2, 2 -diphenyl -1-picrylhydrazyl) scavenging activity [17]. A methanol DPPH solution (0.16%) was mixed with serial dilutions (10–100 μg/mL) of crude extract and after 30 min, the absorbance was read at 515 nm. The radical scavenging activity was expressed as IC_50_ (μg/mL), (the dose required to cause a 50% inhibition).Vitamin C was used as the standard and the ability to scavenge the DPPH radical was calculated by the following formula:

$$\text{DPPH radical scavenging activity }\%=\frac{A_{0}-A_{1}}{A_{0}}\times 100$$

Where A_o_ is the absorbance of the control at 30 min and A_1_ is the absorbance of the sample at 30 min. The experiment was performed in triplicates.

#### ABTS free radical scavenging assay

An ethanolic ABTS [2, 2′-Azino-bis (3-ethylbenzthiazoline-6-sulfonic acid)] solution was mixed with serial dilutions (10–100 μg/mL) of crude extract and after 5 min; the absorbance was recorded at 734 nm [18]. The radical scavenging activity was expressed as IC_50_ (μg/mL), (the dose required to cause a 50% inhibition). Trolox was used as the standard. The ability to scavenge the ABTS radical was calculated by the following formula:

$$\text{ABTS radical scavenging activity }\%=\frac{A_{0}-A_{1}}{A_{0}}\times 100$$

Where A_o_ is the absorbance of the control at 5 min and A_1_ is the absorbance of the sample at 5min. The experiment was performed in triplicates.

### Effect of crude extract on normal cell viability

Viability of L6, H9c2 and RAW 264.7 were assessed by 3-(4, 5-dimethythiazol- 2-yl)-2, 5-diphenyl tetrazolium bromide (MTT) assay [19]. Each cells were seeded (1 × 10^4^ cells/ well) in a 96-well plate. The cells were treated with various concentrations of crude extracts. After 24 h incubation, cells were washed and 100 μl of MTT (5 mg/mL), dissolved in Dulbecco’s modified Eagle’s medium (DMEM), was added to each well and incubated at 37°C in a CO_2_ incubator. After 4 h incubation, DMSO was added to each well and the plate was kept on a shaker at 12 rpm for 45 min. The change in colour was monitored using a micro-plate reader (BIOTEK, USA) at 570 nm. Results were expressed as percentage of cytotoxicity:

$$\text{Percentage of Toxicity}=\frac{\text{Absorbance of Control}-\text{Absorbance of Sample}}{\text{Absorbance of Control}}\times 100$$

### Optimization of pH, temperature and incubation time for maximum antibacterial metabolite production

#### Optimization of temperature

The effect of culture conditions on the production of enhanced antibacterial metabolite production was studied on ISP4 against *S*. *epidermidis* (Pathogenic bacteria which exhibited maximum activity in screening studies). The optimum temperature for the maximum antibacterial compound production was investigated on ISP4. Ten mL bacterial suspension was introduced in 250 mL Erlenmeyer flasks containing 100 mL of broth and incubated at different temperatures (25, 30, 35, 40 and 45°C) at pH 7.0. The antibacterial activity was assayed after 7 days by disk diffusion method as mentioned above.

#### Optimization of pH

The impact of pH on antibacterial metabolite production was studied at different pH, ranges from 5.0 to 12.0 at 35°C and for 7 days.

#### Optimization of incubation period

The optimization of incubation period was also carried out by incubating for 3–15 days at 35°C and pH 7. The antibacterial activity was monitored at every 24 h intervals by disk diffusion method.

### Optimization of medium components in ISP4 for maximum production of antibacterial compounds by response surface methodology using Plackett-Burman design (PBD)

The Plackett—Burman experimental design [20] was chosen for initial evaluation of ISP4 medium components to short-list components having significant effect on antibacterial activity by NIIST A30. The factors considered were macronutrients in ISP4 medium such as starch, K_2_HPO_4_, (NH_4_)_2_SO_4_, CaCO_3_, MgSO_4_ and NaCl, each analysed at two levels, low (-) and high (+). The micronutrients were taken as per standard medium and the total numbers of experiments as per the PBD were 13.The experiments were carried out in triplicates to assess the consistency of results and the average antibacterial activity against *S*. *epidermidis* was determined as the response. The experimental design was developed using the statistical software package, MINITAB 17.

### Optimization of significant components by response surface methodology (RSM)

Based on the preliminary Plackett-Burman analysis, according to the low *p*-values (<0.05) and high confidence levels (>90%), the significant media components were selected as the variables to test in the 15-run experiment of the Box-Behnken design [21]. The variables were studied at low, middle and high concentration levels and were designated as −1, 0 and +1 (coded values), respectively. The MINITAB statistical (MINI-TAB 17) software was used to compute the results and generate response surface graphs.

In this system, the regression analysis was performed to estimate the response function as a second order polynomial equation, Y = β_0_ +∑ β_i_X_i_ + ∑β_ij_X_i_X_j_ + ∑β_ii_X_i_^2^ [22]. Where, Y denotes the predicted response, β_0_ is the intercept term, β_i_ is the linear coefficient, β_ij_ is the quadratic coefficient and β_ii_ is the interaction coefficient, and X_i_X_j_ denotes independent variables under study. The statistical competence of the model was resolved through analysis of variance (ANOVA). The excellence of the polynomial model equation was concluded statistically through coefficient of determination (R^2^) and adjusted R^2^.Three-dimensional response surface plots were produced to elucidate the relationship between the responses and the experimental levels of each independent variable. The optimum level of the variables for maximum antibacterial activity was resolved by response optimizer tool of the software.

### Validation of statistical model and optimization through wet lab

The mathematical model and the optimization were experimentally validated by culturing NIISTA30 under unoptimized and optimized levels of variables at pH 7.0 and 35 ± 2°C for 7 days. After incubation, the antimicrobial metabolite was extracted twice with equal volume of ethyl acetate and dried extract (350 mg/L) was suspended in 100 μl methanol and assayed as above for antibacterial activity.

### Auto biographic test

The ethyl acetate crude extract was evaluated for antibacterial activity through autobiographic agar overlay method [23]. For this, the developed TLC plate was air dried and encased in a sterile Petriplate and overlaid with nutrient agar medium containing 0.6% agar inoculated with test bacterial suspension. The plates were then incubated at 37°C and zone of inhibition around the spots were observed after 24 h.

### Statistical analysis

All data regarding antibacterial activity were subjected to statistical analysis using SPSS software package (Windows version 20.0; IBM SPSS) and expressed as mean± SD. Graphs were plotted using a computer program Origin Pro 8 (Origin Lab, Corporation, USA)

## Results

### Site, sample collection and isolation of *Streptomyces* strains

From the soil samples collected from the different parts of Nelliyampathy Forest site, 140 morphologically different *Streptomyces* strains were isolated. The strains were checked for their purity in AIA and PDA plates and stored on PDA slants, as PDA slants shown better shelf life organisms than AIA and ISP4.

### Antibacterial studies using live *Streptomyces* strains

In primary screening, 16 strains recorded activity against both the indicator test organisms, *S*. *aureus and E*. *coli*. Sixty-eight strains exhibited antibacterial activity against *S*. *aureus* whereas 56 strains showed activity against *E*. *coli*. Out of these strains, NIIST A30 displayed maximum activity against both indicator pathogens (44 and 40 mm against *S*. *aureus* and *E*. *coli* respectively).

Secondary screening was done with 16 strains against eleven pathogenic bacteria of which NIIST A30 exhibited maximum zone of inhibition against all the test pathogens (data not shown). *S*. *epidermidis* was the most inhibited (56 mm) whereas *S*. *typhi* (33 mm) was the least inhibited (Table 1). Thus, the strain NIIST A30 was selected for further detailed investigations.

**Table 1:** *In vitro* antibacterial activity of NIIST A30 against test pathogens.

Test pathogen	Zone of inhibition (mm)
*Bacillus cereus*	41±1.15*
*Bacillus subtilis*	38±0.57
*Escherichia coli*	42±2
*Klebsiella pneumoniae*	43±1
*Mycobacterium smegmatis*	32±2.5
*Pseudomonas aeruginosa*	49±1.15
*Proteus mirabilis*	53±1.15
*Salmonella typhi*	29±1.15
*Staphylococcus aureus*	49±1.15
*Staphylococcus epidermidis*	59±1.15
*Staphylococcus simulans*	54±1.73

* Values are average from three readings.

### Morphological, cultural and physiological characterization

**Fig 1:**
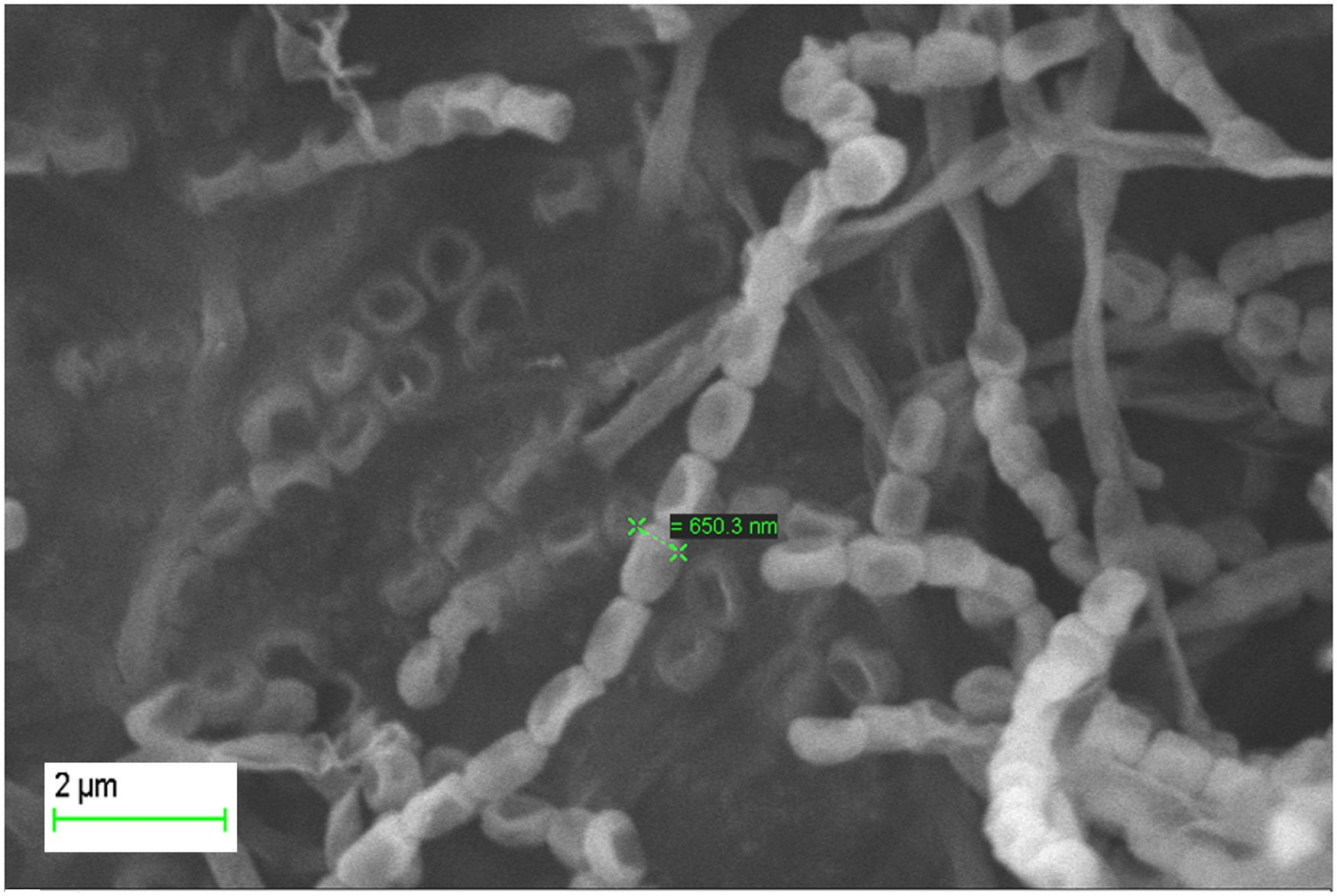
Scanning electron microscopic image of spore arrangement.

The data regarding morphological, cultural and physiological characterization was as shown in supplementary attachments (see Table A, Table B, Table C and Table D in S1 File). Scanning electron microscopic examination showed that the strain formed extensively branched, non- fragmented substrate and arial mycelia. The spore chain morphology of the strain was straight and rectiflexible with smooth surface with less than 50 spores in a chain (Fig 1).

### Molecular characterization NIIST A30

**Fig 2:**
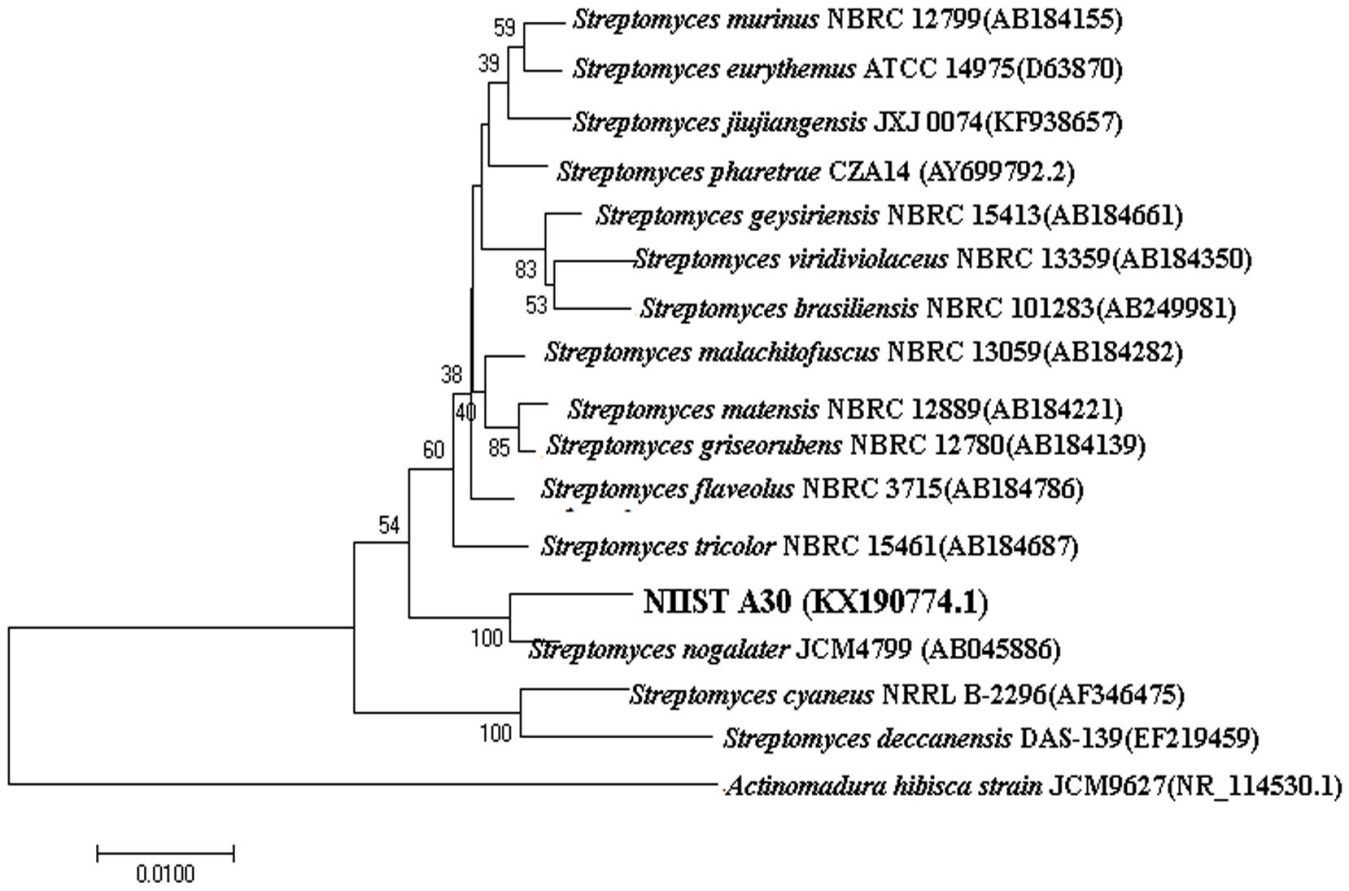
Phylogenetic tree showing the evolutionary relationship of the strain *Streptomyces nogalater* NIIST A30 with its related species.

Genomic DNA isolated from the strain was amplified using universal primers for 16S rRNA. The PCR product showed 1500 bp band on gel electrophoresis was sequenced. The high quality sequence was aligned using Clustal W software. Sequence similarity was done by using Ez taxon database. The 16S rRNA partial sequence was deposited in GenBank data library with an accession No. KX190774.1.The phylogenetic tree was drawn with the help of Mega 6.0 Version software using neighbour joining method. Phylogenetic analysis showed that the strain is identified as *Streptomyces nogalater* with 100% similarity (Fig 2).

### Standardization of optimal fermentation media for significant antibacterial metabolite activity

Among the eight media tested, maximum metabolite production (180 mg/L) and antibacterial activity was recorded in ISP4 followed by glucose soybean meal and yeast malt broth (Table 2).

**Table 2:** Antibacterial activity of NIIST A30 crude extracts from different broths.

Test pathogens	Zone of inhibition(mm)							
	GSMB	ISP 1	ISP 2	ISP 4	KB	NB	SDB	SCB
*B*.*cereus*	9±0.57*	8±0.57	7.6±0.57	**13.6±0.57**	11±0	7±1	8±0	8±0.57
*B*.*subtilis*	10±0	10±0	17.6±0.57	**12±1**	16±0	0	17±0	12.3±0.57
*E*.*coli*	7.6±0.28	7.5±0.5	9±0	**10±0**	8±0.57	9.3±0.57	6±0	10±0
*K*.*pneumoniae*	6.5±0.5	8.3±0.57	9±1	**12±0**	11±0	10.3±0.57	7±0	10.3±0.57
*M*.*smegmatis*	11±1	10±1	10±1	**11±0**	11.3±0.28	6±0	6±0	9.16±0.28
*P*.*aeruginosa*	9.3±0.57	0	7.6±0.57	**10±1**	6.8±0.28	0	8±0.57	9±1
*P*.*mirabilis*	7.5±	0	8±0.57	**11±0**	10±0	10±0	11±0	10.3±0.28
*S*.*aureus*	9.3±0.28	10±0	10±0	**9±0.57**	8±0.57	7±0	8±0	10±0
*S*.*epidermidis*	13.3±0.28	12.3±0.28	11±1	**15±1.52**	14±0.57	12±0	13.3±0.28	14±0.57
*S*.*simulans*	10±0	11±0	6±1	**12±1**	10±0	12±0	7±0	8±0.57
*S*.*typhi*	10±1	5±0	9±0	**12±1**	8±0	10±0	7.6±0.57	5±0

*Values are average from three readings.

### Antioxidant activities of crude ethyl acetate extract (scavenging of free radicals)

**Fig 3:**
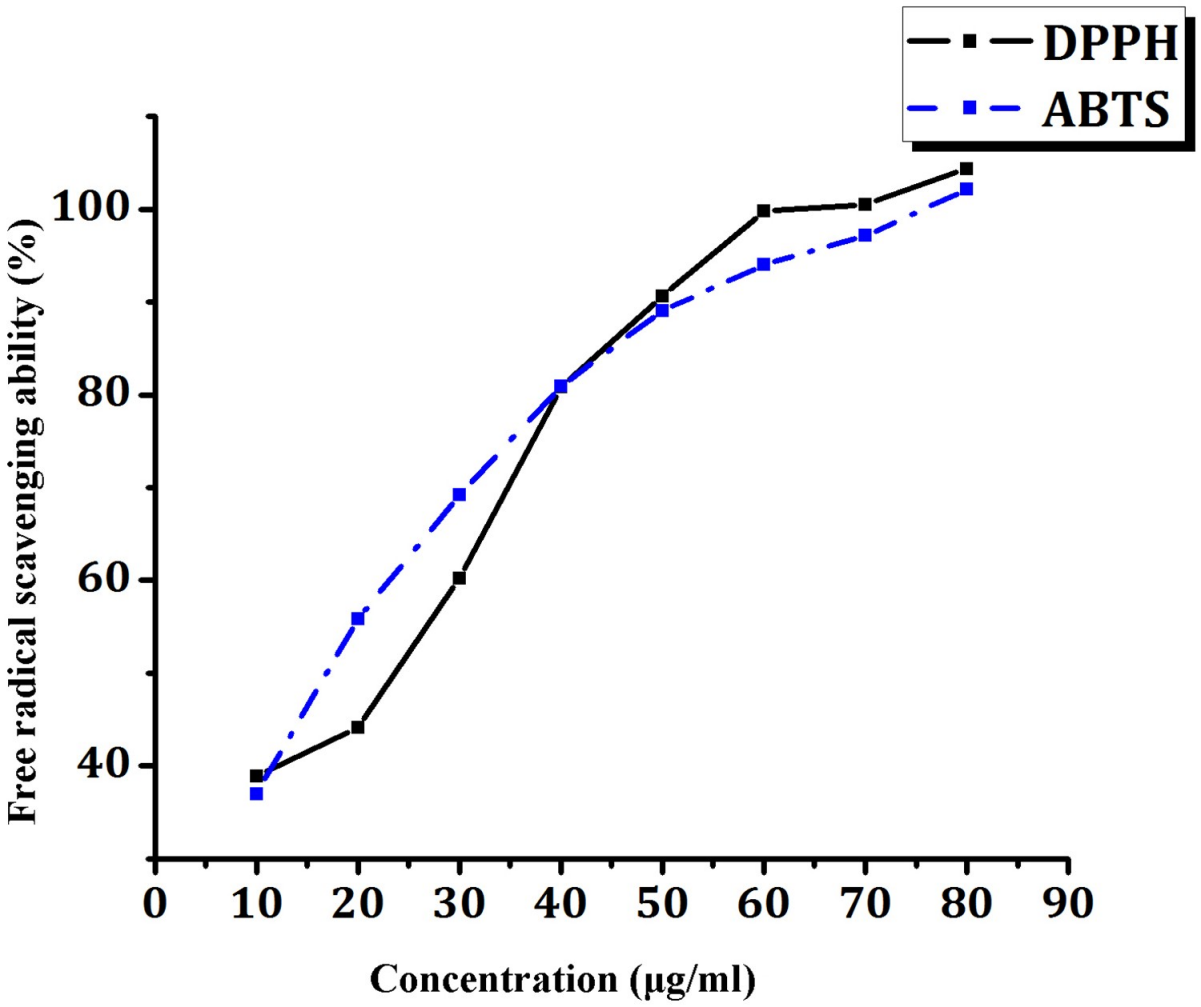
Free radical scavenging activity of ethyl acetate crude extract.

Crude extract exhibited significant dose dependent inhibition of DPPH activity, with a 50% inhibition (IC_50_) at 30 μg/mL. The IC_50_ value for vitamin C was 295 μg/mL. ABTS scavenging activity demonstrates 50% inhibition (IC_50_) at 20 μg/mL (Fig 3).

### Effect of crude extract on normal cell viability

**Fig 4:**
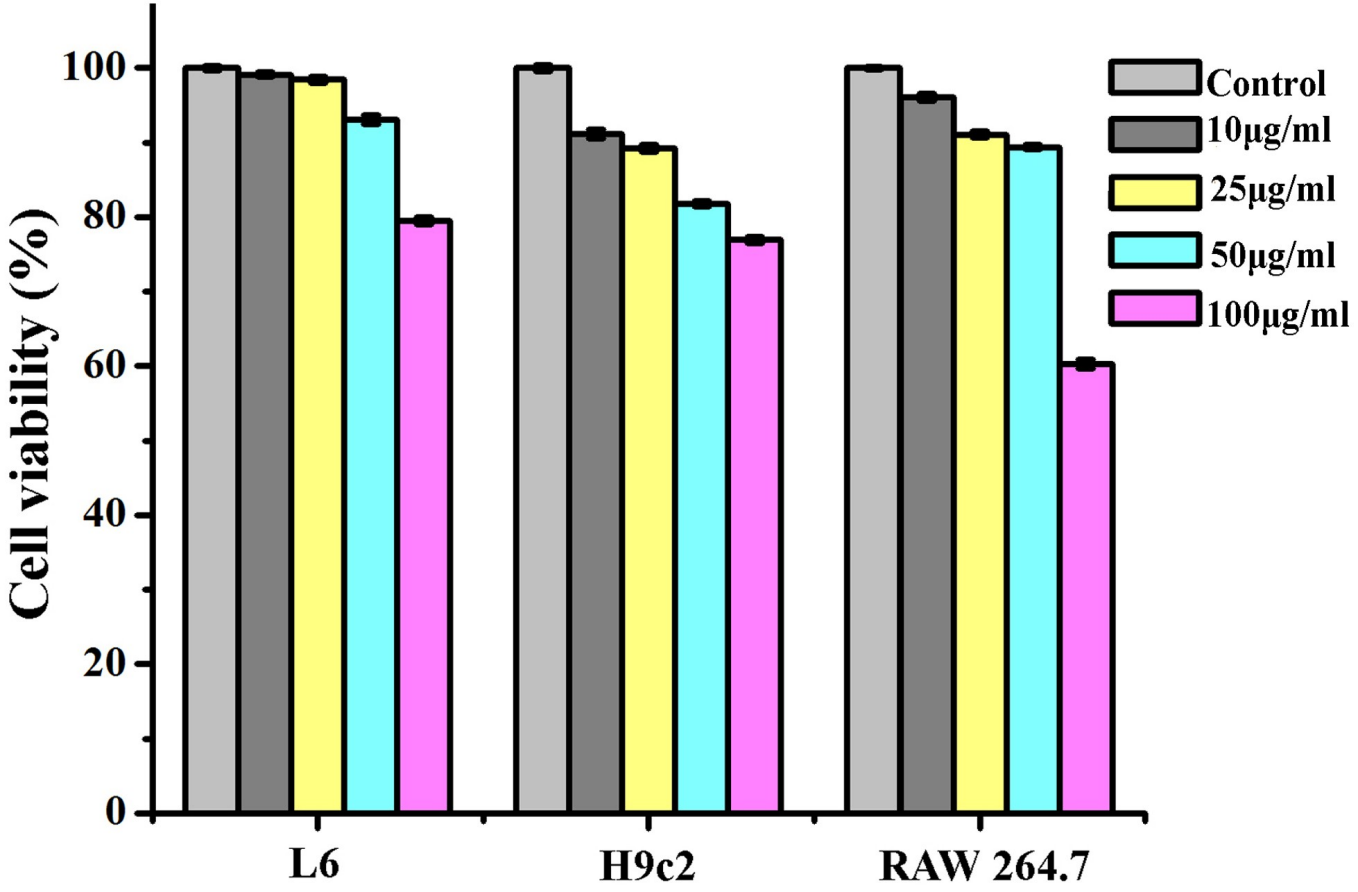
The effect of crude extract on normal cell line viabilities.

The concentration of crude extract up to 50 μg/mL was found to be less than 20% toxic in all the cell lines for a period of 24 h (Fig 4). However, a concentration of 100 μg/mL and above was found to be toxic (40%) in RAW 264.7 cell lines whereas in L6 and H9c2 cell lines the extract exhibited 20 and 23 percentages toxicity, respectively.

### Optimization of fermentation parameters for maximum antibacterial activity

**Fig 5:**
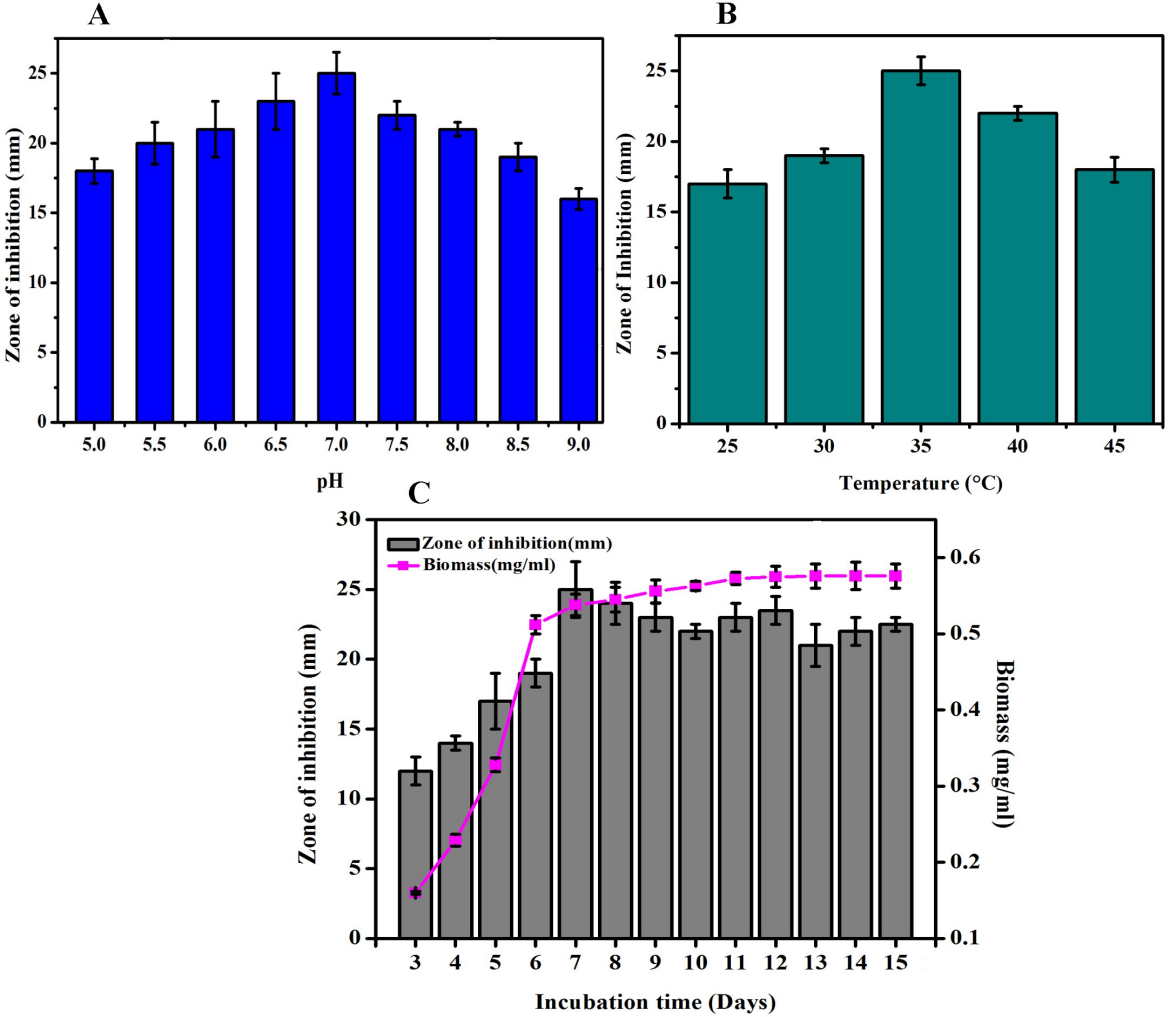
Antimicrobial activity of NIIST A30 in different pH (A), temperature (B) and incubation period (C).

The strain exhibited maximum antibacterial activity against *S*. *epidermidis* at pH 7.0 (Fig 5A) at a temperature of 35°C (Fig 5B). Above and below these conditions, a decreased level of antibacterial production was recorded by the organism. The strain exhibited antibacterial activity from 3^rd^day onwards which attained maximum on 7^th^ day and declined for the rest of the period (Fig 5C).

### Allocation of essential medium components using Placket-Burman design

**Fig 6:**
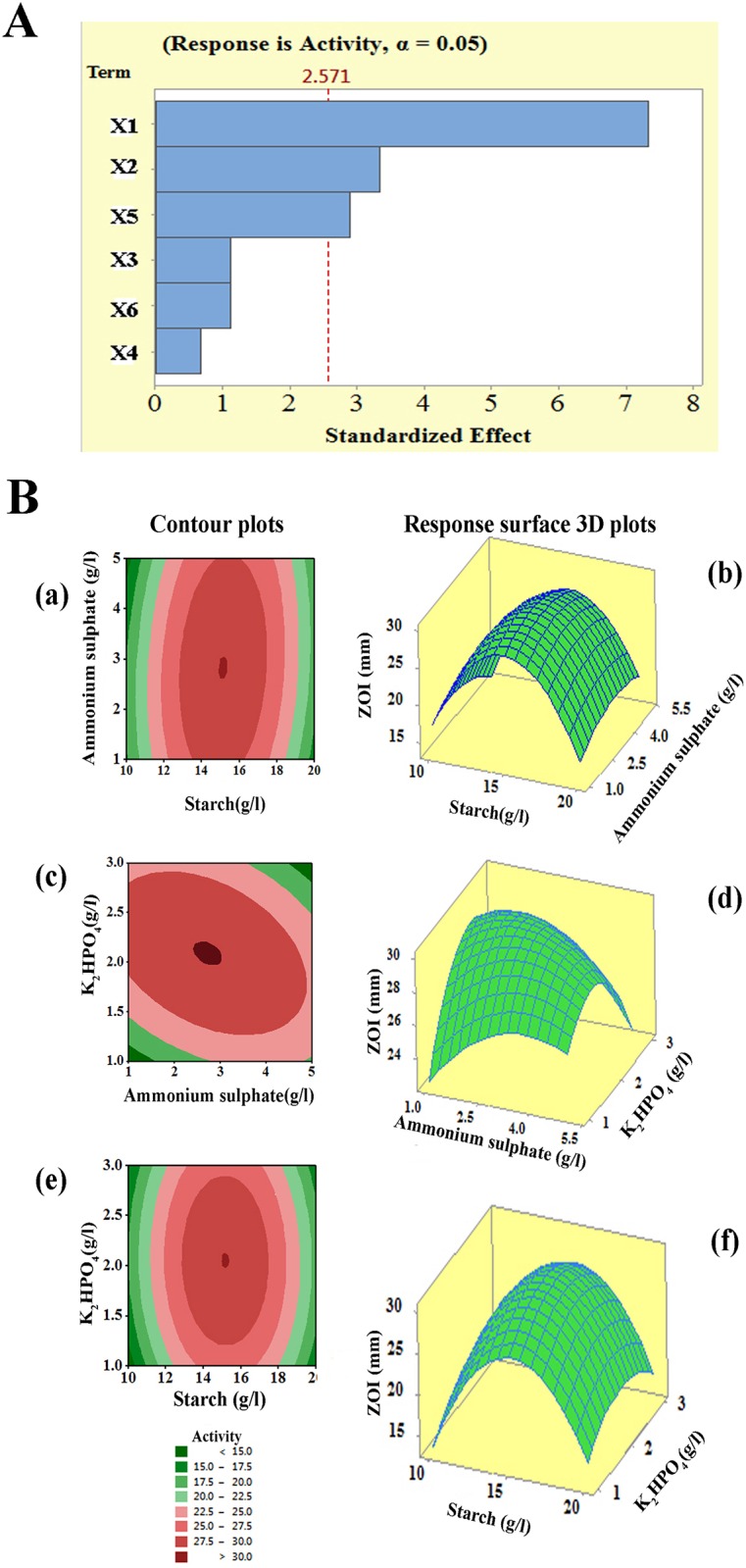
(A) Pareto chart showing the effect of different variables (media components) on antibacterial metabolite production against *S*. *epidermidis*; (B) Response surface contour and 3D plots showing individual and interactive effects of variables on antibacterial activity of NIIST A30. (a) and (b) Effects of (NH_4_)_2_SO_4_ and starch on antimicrobial activity(c) and (d) Effects of K_2_HPO_4_ and (NH_4_)_2_SO_4_ on antimicrobial activity (e) and (f) Effects of K_2_HPO_4_ and starch on antimicrobial activity.

Six media components were analyzed for their impact on antibacterial activity against *S*. *epidermidis* using Placket-Burman design with 13 experiment set up to observe significant media components (Tables 3 and 4). Effect estimates and analysis of variables for antibacterial activity from experimental design was as shown in Table 5. Based on the low *p*-values and high confidence levels, 3 variables such as starch, (NH_4_)_2_SO_4_ and K_2_HPO_4_ were determined to have a significant effect on the antibacterial compound production. Pareto chart (Fig 6A) strongly confirm the fact that the most important factors influencing antibacterial compound production were starch, (NH_4_)_2_SO_4_ and K_2_HPO_4_.

**Table 3:** Range of variables used for PBD.

Factor codes	Factors	Levels	
		-1 (g/L)	+1 (g/L)
X_1_	Starch	7	13
X_2_	K_2_HPO_4_	0.5	1.5
X_3_	MgSO_4_.7H_2_O	0.5	1.5
X_4_	NaCl	0.5	1.5
X_5_	(NH_4_)_2_SO_4_	1	3
X_6_	CaCO_3_	1	3

**Table 4:** Plackett-Burman experimental design.

Run	Factors						Antibacterial Activity against *S*. *epidermidis*, ZOI (mm)
	X_1_	X_2_	X_3_	X_4_	X_5_	X_6_	
1	7	0.5	0.5	0.5	1	1	12 ± 1
2	13	0.5	1.5	0.5	1	1	21 ± 0.5
3	7	0.5	0.5	1.5	3	3	17 ± 1.5
4	7	1.5	1.5	1.5	1	3	18 ±1
5	7	1.5	1.5	0.5	3	1	19 ± 0.5
6	13	1.5	0.5	1.5	3	1	25 ± 1
7	13	0.5	1.5	1.5	1	3	21 ± 0.5
8	13	1.5	1.5	0.5	3	3	24 ± 1.5
9	13	0.5	0.5	0.5	3	3	22 ± 1
10	10	1	1	1	2	2	20 ± 0.5
11	7	1.5	0.5	0.5	1	3	18 ± 2
12	7	0.5	1.5	1.5	3	1	17 ± 1.5
13	13	1.5	0.5	1.5	1	1	21 ± 1

**Table 5:** Statistical analysis of effects of variables (media components) on antibacterial activity employing Plackett-Burman design.

Variables	Medium constituents	Effect	*t*-value	*p*- value *	Confidence level (%)	Standard error coefficient
X_1_	Starch	1.833	7.34	**0.001**	**99.9**	0.125
X_2_	K_2_HPO_4_	5.000	3.34	**0.021**	**97.9**	0.749
X_3_	MgSO_4_.7H_2_O	1.667	1.11	0.317	68.3	0.749
X_4_	NaCl	1.000	0.67	0.534	41.6	0.749
X_5_	(NH_4_)_2_SO_4_	2.167	2.89	**0.034**	**96.6**	0.375
X_6_	CaCO_3_	0.833	1.11	0.317	68.3	0.375

*: at confidence level of 95%, values ≤ 0.05 is acceptable.

### Optimization of selected media components using Box-Behnken design

The variables which give a *p*- value less than or equal to 0.05 were chosen for next level of optimization by response surface methodology with Box-Behnken design. The significant media components such as starch, (NH_4_)_2_SO_4_ and K_2_HPO_4_ were selected recorded response as antibacterial activity and the design matrix was as shown in Table 6. The regression equation coefficients were also determined and the data was fitted to a second-order polynomial equation. The RSM response regarding antibacterial activity is expressed as the following equation:

$$\text{Y}=-98.56+13.575{\text{X}}_{1}+2.937{\text{X}}_{5}+18.12\;{\text{X}}_{2}-0.4700\;{\text{X}}_{\text{1}}{\text{X}}_{\text{1}}-0.5000\;{\text{X}}_{\text{5}}{\text{X}}_{5}-3.500\;{\text{X}}_{\text{2}}{\text{X}}_{\text{2}}+0.1250\;{\text{X}}_{\text{1}}{\text{X}}_{\text{5}}-0.0500{\text{X}}_{\text{1}}{\text{X}}_{\text{2}}-1.000\;{\text{X}}_{\text{5}}{\text{X}}_{\text{2}}.$$

Where Y is the antibacterial activity (zone of inhibition in mm) against *S*. *epidermidis* and X_1_, X_2_ and X_5_ were starch, K_2_HPO_4_, and (NH_4_)_2_SO_4_ respectively. The statistical significance of the fitted model was evaluated by ANOVA (Table 7). The predicted R^2^ of 96.48% is in reasonable agreement with the adjusted R^2^ value of 99.38%.

**Table 6:** Box-Behnken response surface design.

RunOrder	Starch	(NH_4_)_2_SO_4_	K_2_HPO_4_	Antimicrobial Activity ZOI (mm)
1	15	5	1	26 ± 1.5
2	15	3	2	**30 ± 2**
3	20	3	1	15 ± 1
4	15	5	3	22 ± 1.5
5	20	5	2	18 ± 1
6	10	3	1	13 ± 1.5
7	15	1	3	27 ± 0.5
8	20	3	3	16 ± 1
9	15	3	2	**30 ± 1.5**
10	10	1	2	17 ± 0.5
11	15	1	1	23 ± 1
12	10	3	3	15 ± 1
13	20	1	2	16 ± 0.5
14	15	3	2	**30 ± 1.5**
15	10	5	2	14 ± 1

**Table 7:** Analysis of variance of fitted quadratic model.

Source	Degrees of freedom	Sum of Squares	Mean Sum of squares	*F*-Value	*p*-Value
Model	9	567.150	63.017	252.07	0.000
Error (Residual)	5	1.250	0.250		
Lack-of-Fit	3	1.250	0.417		
Pure Error	2	0.000	0.000		
Total	14	568.400			

Determination of co-efficient, R^2^ = 0.9648; Adjusted determination of co-efficient, Adj R^2^ = 0.9938.

### Response surface or contour plots

By using the response surface 3D plots (Fig 6B), the interactions between the two factors and their optimum levels were studied. Fig 6B(a) and 6B(b) shows the effect of starch and (NH_4_)_2_SO_4_ on antibacterial activity. With a moderate concentration of starch and (NH_4_)_2_SO_4_, the antibacterial activity increased and after that the activity decreased with a higher concentration of starch and (NH_4_)_2_SO_4_. The same trend was observed in the effects of K_2_HPO_4_ and starch on antibacterial activity [Fig 6(c) and 6(d)]. Fig 6B(e) and 6B(f) shows the effect of K_2_HPO_4_ and (NH_4_)_2_SO_4_ on antibacterial activity. Initially, the antibacterial activity increased with increasing concentration of both K_2_HPO_4_ and (NH_4_)_2_SO_4_. Further addition of K_2_HPO_4_ and (NH_4_)_2_SO_4_ leads to reduced antibacterial activity. The 3-Dimensional RSM plots clearly indicated that the maximum antibacterial activity should appear with a medium level of starch, K_2_HPO_4_ and (NH_4_)_2_SO_4_. With the help of numerical optimization, the quadratic model predicted that the optimal values of test factors viz, starch = 14.97 g/L, (NH_4_)_2_SO_4_ = 2.89 g/L, and K_2_HPO_4_ = 2.07 g/L should give a maximum antibacterial activity of 30 mm zone of inhibition.

### Experimental validation of optimization

The medium component parameters predicted from RSM was experimentally proven in triplicates. The antibacterial activity against *S*. *epidermidis* obtained experimentally was 28.5 ± 1.5mm which was in close accordance with the predicted value of 30 mm. The final optimized medium contained 14.97 g of soluble starch, 2.89g of (NH_4_)_2_SO_4_, 2.07g of K_2_HPO_4_, 1g of MgSO_4_·7H_2_O, 1g of NaCl, 2g of CaCO_3_, 1mg of FeSO_4_.7H_2_O, 1 mg of MnCl_2_.7H_2_O and 1mg of ZnSO_4_.7H_2_O as the initial concentration per liter of distilled water.

### Bioautography in thin layer chromatography

The TLC bioautography proved the antibacterial activity of compounds against *S*. *epidermidis* with particular reference to compound 1 (R_f_ 0.65) and compound 2 (R_f_ 0.3). Compound 2 was exhibited prominent antibacterial activity (13±0.57 mm) against the pathogen as compared to compound 1 (5±0.57 mm).

## Discussion

*Streptomyces* is ubiquitous in soil microbial communities and more than 500 species have been already described [24]. The members of *Streptomyces* species are renowned for their ability to produce bioactive secondary metabolites and act as a main source for secondary metabolites. Considering the demand for new antibiotics, the present study was aimed to characterize various *Streptomyces* species from Nelliyampathy forest, an integral part of Western Ghats of India, with novel flora and fauna. The forest area has been preferred because of its unexplored abundance in microbial flora [25]. Isolation of new microbial species from unexplored as well as extreme habitats is one of the efficient strategies for the mining of potential microbial compounds. With this perspective, an antibacterial metabolite producing strain NIIST A30 was recognized as *Streptomyces nogalater* based on 16SrRNA based phylogenetic analysis with 100% sequence similarity.

The emergence of resistance in bacterial pathogens is eroding our capability to contain life threatening infections with the existing drugs. So we need novel drug molecules to combat against drug resistant pathogens. The antimicrobial drug research program worldwide has to be focussed on various drugs against multidrug resistant pathogens. Hence, the current study has been enriching the search of potential microbes with novel antibacterial properties. In the present study, the strain NIIST A30 showed broad spectrum antibacterial activity against Gram positive and negative test bacteria. Sixteen out of 140 isolates exhibited potential antibacterial activity against both types of test bacteria. Sixty eight strains were active against Gram positive bacteria alone while fifty six were found active against Gram negative bacteria alone. The results (48.57% and 40% against Gram positive and negative respectively) are higher than the previous reports on isolation of antibiotic actinomycetes from soils [26, 27]. In a study, Dehand et al. [28], reported the isolation of *Streptomyces* strains with antagonistic activity (13.3%) from soils of West of Iran.

Most of the metabolites produced by *Streptomyces* are extracellular in nature, with potent antimicrobial activities [29]. The enhanced production of secondary metabolites usually involves the choice of appropriate fermentation medium. In this study, out of eight fermentation media tested, inorganic salts starch broth (ISP4) recorded maximum antibacterial activity which indicated that the strain was able to utilize starch for better antibacterial metabolite production. It is known that the presence of carbon sources in fermentation media enhances the production of bioactive secondary metabolites from microbes. Kumar et al. [30] reported the best enhanced antimicrobial metabolite production from a *Bacillus* species, when fructose was used as the carbon source among the water-soluble carbon sources. The current study also indicated that the synthesis of bioactive metabolites varies with the modification of fermentation media.

A source of carbon rapidly assimilable exerts a negative effect on biosynthesis (catabolic repression or ‘‘glucose effect”) [31] which can be overcome by the use of complex carbon source like polysaccharides (starch, dextrins). According to Messis et al. [32], a complex source of carbon metabolizable (starch) increased antifungal production by a *Streptomyces* sp. TKJ2 and Fukuda et al. [33] also used starch as a carbon source in culture medium for improved antifungal production by *Streptomyces* sp. K03-0132. In this study too, *Streptomyces nogalater* NIIST A30 was able to produce an enhanced level of broad spectrum antibacterial metabolites with starch as carbon source.

Cultural parameters also influence the production of bioactive secondary metabolites [34]. In the present study, fermentation conditions such as pH, temperature and incubation time were optimized through conventional means. NIIST A30 showed best growth and antimicrobial activity at pH 7.0, 35°C after an incubation period of 7 days. These results are somewhat similar to the findings of Jose and Debakumar [35], who reported maximum antimicrobial activity of pH 7.5 at 32°C after 11 days with a rare actinomycete, *Nonomuraea* sp. JAJ18. From earlier reports, it is also evident that the maximum antibiotic production by *Streptomyces* cultures required at least 96 h.

In this study, NIIST A30 was investigated for the scavenging abilities on DPPH and ABTS synthetic radicals. DPPH is a useful reagent to evaluate the free radical scavenging ability of the hydrogen donating antioxidant, which can transfer hydrogen atoms or electrons to DPPH radicals. Compounds from NIIST A30 were able to reduce the stable radical DPPH to the yellow coloured diphenylpicrylhydrazine. Hydroxyl radical is one of the reactive oxygen species generated in the body and removing hydroxyl radicals is important for antioxidant defence in living cell systems [36].On the other hand, ABTS radical cation which is blue in colour reacts towards antioxidants and converted to a colourless neutral form. Even though these methodologies present similarities, a considerable antioxidant capacity was observed, and ABTS radical scavenging was higher than DPPH (40% to 80% scavenging). A probable explanation for this phenomenon is the fact that ABTS is soluble in water as well as in organic solvents, allowing the antioxidant activity of hydrophilic and lipophilic compounds [37].The cytotoxicity in normal cell lines demonstrated the non-toxic effect of crude extract up to a concentration of 50 μg/mL, which indicates the non-toxic effect of compounds in the crude extract.

The antibiotic producing capability of *Streptomyces* sp. is not a static property and it can be significantly affected by constituents of production medium. Previous studies on secondary metabolite production were conducted using conventional methods. However, this method frequently failed to measure the region of optimum response because the combined effect of parameters has not been considered. Statistical methods such as RSM have been used in various phases of fermentation optimization [38, 39]. In this study, the significant media components were statistically optimized for enhanced antibacterial production against *S*. *epidermidis*. The compelling media components were screened and selected using Plackett-Burman design. Data obtained showed that the starch, K_2_HPO_4_ and (NH_4_)_2_SO_4_ exerted positive effects rather than other components. Ripa et al. [40] reported that the antibiotic production in *Streptomyces* sp. RUPA-08PR was markedly increased in medium supplemented with K_2_HPO_4_. In another study, Zhu et al. [41] reported the importance of optimal concentration of (NH_4_)_2_SO_4_ in antibiotic production by *Streptomyces viridochromogenes*. In the present study, media components such as starch, (NH_4_)_2_SO_4_ and K_2_HPO_4_ in moderate amount facilitated maximum metabolite production by NIIST A30.

The statistical significance of the model was checked by *p*-value and co-efficient of determination (R^2^). In the current work, the R^2^ value was found to be 0.9648 which means that the model can explain 96.48% of total variations. The closer the R^2^ to 1.0, the stronger the model and better it predicts the response. The model suggested that the starch, (NH_4_)_2_SO_4_ and K_2_HPO_4_ affected the metabolite production from NIIST A30. The response surface and 2D contour plots obtained with Box-Behnken design illustrated the interactive effects of two independent variables while maintaining the third variable at a fixed level.

In the present study, after optimization through RSM, the antibacterial activity has been notably increased when compared to unoptimized medium. Antibacterial activity against *S*. *epidermidis* in terms of zone of inhibition was increased from 15±1.5 to 28±1.5 mm. An 86.66% increase from modified media strongly suggests that the quantity of media components affects antibacterial metabolite production in *S*. *epidermidis*. Hence, the current statistical experimental design was found to be accurate in optimizing the significant media components. It is a first report from a *Streptomyces* sp. where an 86.66% increase in antibacterial activity against *S*. *epidermidis* isolated from a forest soil.

## Conclusions

The strain *Streptomyces nogalater* NIIST A30 recorded a broad spectrum antibacterial activity, of which maximum activity obtained against a clinical pathogen, *S*. *epidermidis*, a causative agent of nosocomial infections. The ethyl acetate crude extract also provided significant antibacterial potential. In the present study, out of eight media tested, ISP4 recorded better antibacterial production. Fermentation medium optimization for maximum antibacterial activity was achieved through statistical methods using RSM in ISP4 broth. The optimization resulted in a maximum antibacterial activity of 28.15±1.5mm which is an 86.66% increase in comparison with that obtained in an unoptimized broth. Thus, the statistical trial using RSM for optimization of medium compounds by *Streptomyces nogalater* NIIST A30 was validated to be a potent and useful tool. Further studies on the purification, characterization and structural elucidation of active compound(s) from the crude metabolites and their *modus operandi* is under progress for its further exploitation.

## Supporting information

S1 File Supporting information file containing Figure A and Tables A-E. (DOCX)
