# Supplementary material for: Enhanced antibacterial metabolite production through the application of statistical methodologies by a Streptomyces nogalater NIIST A30 isolated from Western Ghats forest soil
Source: PLoS One. 2017 Apr 24;12(4):e0175919. doi: 10.1371/journal.pone.0175919 (PMC5402949; doi:10.1371/journal.pone.0175919)
Supplement: S1 File — (DOCX) [file pone.0175919.s001.docx]

**Supporting information (S1)**

Morphological, cultural and physiological characterization of NIIST A30

NIIST A30 produced grey coloured well-developed arial mycelium and spores. The colour of the reverse side of the vegetative mycelium was brown. It showed the production of olive brown soluble pigment in different media. Melanin reaction was positive in ISP 7. The morphological characteristics were different from media to media (Table A). The sporulating and pigment producing ability of the isolate was strongly dependent on the media composition (Fig A).

The strain was Gram positive and aerobic with catalase, oxidase positive. It also exhibited biochemical properties including hydrolytic activity on starch, urea, cellulose, protein and lipids. But it did not possess hydrolytic activity on pectin and gelatin. The strain showed antibiotic susceptibility against seven tested antibiotics (Table B). NIIST A30 utilized various carbon sources except maltose and nitrogen sources except potassium nitrate (Table C and Table D ). Cultural characteristics of the strain showed that it tolerated pH ranges from 0.5 to 12 and was able to grow well at pH 2 to 9. The isolate was able to grow well at temperatures ranges from 25°C to 45°C.

Fig A: Morphology and growth of NIIST A30 in various media.


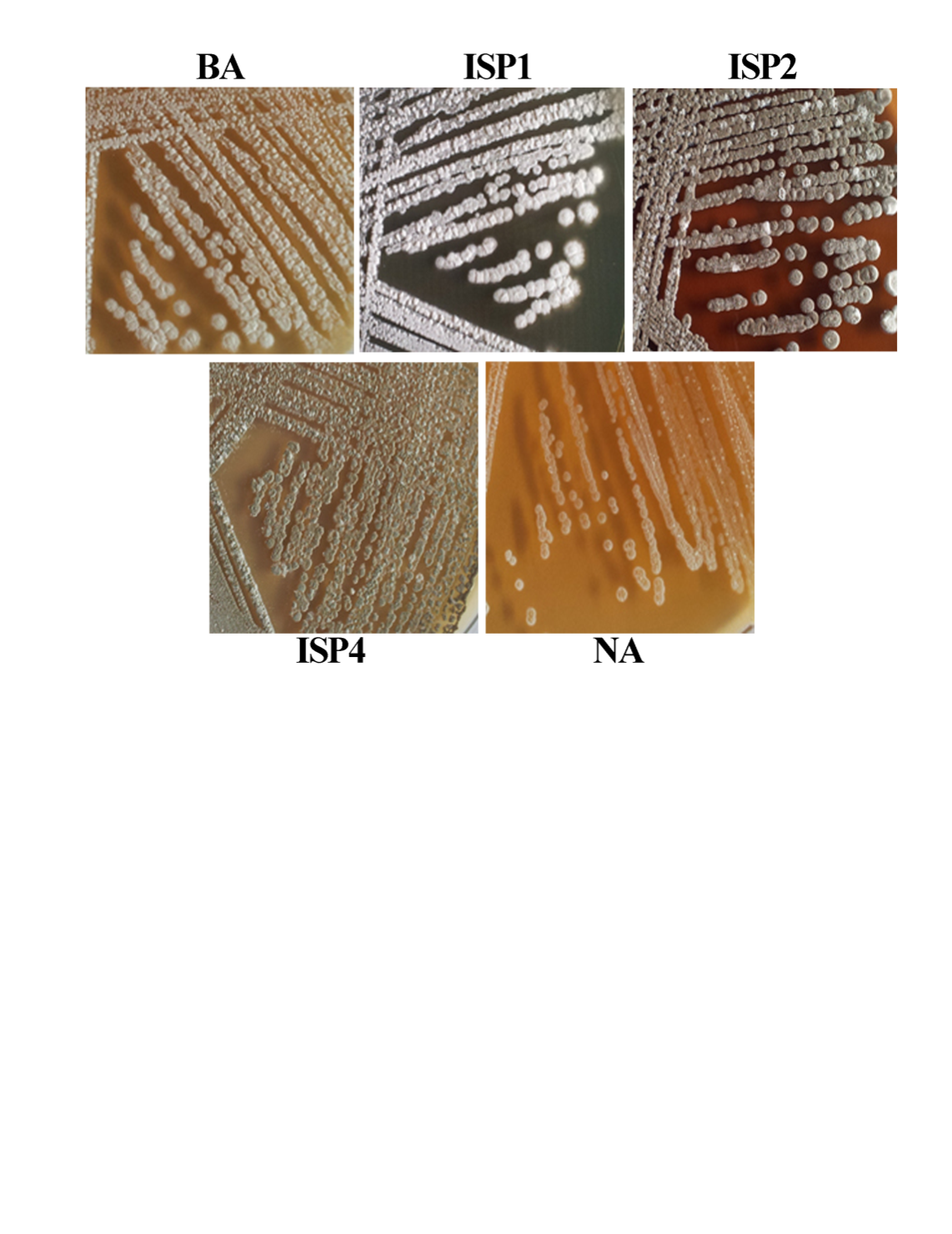


Table A: Morphological characteristics of NIIST A30 in different media.

| **Media** | **Growth** | **Arial mycelium**  **(color)** | **Substrate mycelium**  **(Color)** | **Pigmentation**  **(Color)** | **Sporulation** |
| --- | --- | --- | --- | --- | --- |
| AIA | Poor | White | Grey, not fully develop | Nil | Nil |
| BA | Good | White | White to grey | Nil | Nil |
| CDA | Poor | Grey | Poorly develop | Nil | Nil |
| ISP 1 | Moderate | White | Not fully develop | Nil | Nil |
| ISP 2 | Very good | Grey | Grey | Olive brown | Good |
| ISP 3 | Less | Grey | Dark yellow | Deep yellow | Less |
| ISP 4 | Good | Immature white | Not fully develop | Nil | Good |
| ISP 5 | Good | Grey | Strong yellowish brown | Deep brown | Good |
| ISP 6 | Less | Grey | Strong yellowish | Moderate olive brown | Good |
| ISP 7 | Moderate | Grey | Moderate yellow | Dark yellow | Good |
| KA | Moderate | White to grey | Deep yellowish brown | Deep brown | Less |
| NA | Moderate | white | Not fully develop, white | Nil | Less |
| PDA | Very good | White | Brown, well develop | Olive brown | Good |
| SCA | Moderate | Grey | Dark brown | Deep brown | Less |

Table B: Antibiotic susceptibility for strain NIIST A30

| Antibiotics Antibiotics | | | |
| --- | --- | --- | --- |
| Doripenem | - | Chloramphenicol | + |
| Kanamycin | - | Ciprofloxacin | + |
| Cinoxacin | - | Carbenicillin | + |
| Nalidixic Acid | - | Piperacillin | _+_ |
| Minocycline | - | Clindamycin | + |
| Minonazole | - | Imipenem | + |
| Fluconazole | + | Methicillin | - |
| Methicillin | + | Rifampicin | + |
| Nafcilin | + | Ampicillin | + |
| Ketoconazole | + | Clotrimazole | + |
| Nystatin | + | Amphotericin B | + |

Table C: Utilization of different carbon sources

| Carbon source |  | Growth^#^ |
| --- | --- | --- |
| Fructose |  | ++ |
| Galactose |  | + |
| Glucose |  | + |
| Glycerol |  | ++ |
| Lactose |  | + |
| Maltose |  | - |
| Mannitol |  | + |
| Starch |  | +++ |
| Sucrose |  | + |
| Xylose |  | + |

**#**: **+++**, excellent growth; **++** moderate growth;

**+**, poor growth; **-**, no growth.

Table D: Utilization of different nitrogen sources

| Nitrogen source | Growth^#^ |
| --- | --- |
| Ammonium chloride | ++ |
| Ammonium sulphate | +++ |
| Beef extract | ++ |
| Biopeptone | + |
| Casein | + |
| Malt extract | ++ |
| Meat infusion | + |
| Meat peptone | ++ |
| Peptone | +++ |
| Potassium nitrate | - |
| Sodium citrate | + |
| Soyabean meal | +++ |
| Urea | + |
| Yeast extract | ++ |

**#**: **+++**, excellent growth; **++** moderate growth;

**+**, poor growth; **-**, no growth.

| **Glucose soybean meal broth** | | **Tryptone yeast extract broth** | | **Yeast malt broth** | | **Inorganic salt starch broth** | |
| --- | --- | --- | --- | --- | --- | --- | --- |
| Glucose | 10.0g | Casein enzymic hydrolysate | 5.0g | Yeast extract | 4.0g | Starch soluble | 10.0g |
| Soyabean meal | 10.0g | Yeast extract | 3.0g | Malt extract | 10.0g | K_2_HPO_4_ | 1.0g |
| NaCl | 10.0g | Distilled water | 1L | Dextrose | 4.0g | MgSO_4_.7H_2_O | 1.0g |
| CaCO_3_ | 1.0g |  |  | Distilled water | 1L | NaCl | 1.0g |
| Distilled water | 1L |  |  |  |  | (NH_4_)_2_SO_4_ | 2.0g |
|  |  |  |  |  |  | CaCO_3_ | 2.0g |
|  |  |  |  |  |  | FeSO_4_. 7H_2_O | 0.001mg |
|  |  |  |  |  |  | MnCl_2_ | 0.001mg |
|  |  |  |  |  |  | ZnSO_4_ | 0.001mg |

Table E: Composition of media used for the selection of production medium.

| **Kuster’s broth** | | **Nutrient Broth** | | **Sabouraud dextrose broth** | | **Starch casein broth** | |
| --- | --- | --- | --- | --- | --- | --- | --- |
| Glycerol | 1.0g | Peptone | 5.0g | Dextrose | 20.0g | Starch | 10.0g |
| Casein | 0.3g | Beef extract | 1.5g | Meat peptone | 10.0g | K_2_HPO_4_ | 2.0g |
| KNO_3_ | 2.0g | Yeast extract | 1.5g | Distilled water | 1L | KNO_3_ | 2.0g |
| K_2_HPO_4_ | 2.0g | NaCl | 5.0g |  |  | Casein | 0.3g |
| Soluble starch | 0.5g | Distilled water | 1L |  |  | MgSO_4_.7H_2_O | 0.05g |
| Asparagine | 0.1g |  |  |  |  | CaCO_3_ | 0.02g |
| FeSO_4_. 7H_2_O | 0.01g |  |  |  |  | FeSO_4_. 7H_2_O | 0.01g |
| CaCO_3_ | 0.02g |  |  |  |  | Distilled water | 1L |
| MgSO_4_.7H_2_O | 0.05g |  |  |  |  |  |  |
| Distilled water | 1L |  |  |  |  |  |  |
